# Changes in Serum Proteins in Cats with Obesity: A Proteomic Approach

**DOI:** 10.3390/ani15010091

**Published:** 2025-01-03

**Authors:** Esmeralda Cañadas-Vidal, Alberto Muñoz-Prieto, Dina Rešetar Maslov, Ivana Rubić, Juan C. González-Sánchez, Juan D. Garcia-Martinez, José J. Ceron, Vladimir Mrljak, Luis Pardo-Marin, Silvia Martinez-Subiela, Asta Tvarijonaviciute

**Affiliations:** 1 Interdisciplinary Laboratory of Clinical Analysis Interlab-UMU, Regional Campus of International Excellence ‘Campus Mare Nostrum’, University of Murcia, Campus de Espinardo s/n, Espinardo, 30100 Murcia, Spain; esmeralda.canadas@um.es (E.C.-V.); jjceron@um.es (J.J.C.); lpm1@um.es (L.P.-M.); silviams@um.es (S.M.-S.); asta@um.es (A.T.); 2 Veterinary Clinical Hospital, University of Murcia, Calle Campus Universitario 16, Espinardo, 30100 Murcia, Spain; 3 Department of Animal Medicine and Surgery, Faculty of Veterinary Medicine, University of Murcia, Calle Campus Universitario, Espinardo, 30100 Murcia, Spain; juandi@um.es; 4 Internal Diseases Clinic, Faculty of Veterinary Medicine, University of Zagreb, 10000 Zagreb, Croatia; drmaslov@vef.unizg.hr (D.R.M.); irubic@vef.unizg.hr (I.R.); vmrljak@vef.hr (V.M.); 5 BioQuant, Heidelberg University, Im Neuenheimer Feld 267, 69120 Heidelberg, Germany; juan-carlos.gonzalez@bioquant.uni-heidelberg.de

**Keywords:** cat, Ig-like domain-containing protein, Alpha-2-HS-glycoprotein, complement, antithrombin, Serpin family A member 1, albumin, C4b-binding protein

## Abstract

Obesity is defined as the excessive accumulation of adipose tissue and is currently the most common disease in cats. Similarly to humans, obesity negatively impacts the health and welfare of cats, predisposing them to many other disorders. The objective of this study was to compare the serum proteomes of normal-weight and overweight/obese cats, aiming to gain insights into the physiopathology of feline obesity and potentially identify new biomarkers. For this, serum samples from a total of 20 adult neutered domestic shorthair client-owned cats, ten normal weight and ten overweight/obese, were submitted to tandem mass tags labelling and liquid chromatography-mass spectrometry (LC-MS/MS) analysis. A total of 288 proteins were detected in the serum samples. Out of these, 12 proteins showed statistically significant differences in abundance between control cats and cats with obesity, namely Ig-like domain-containing protein, Alpha-2-HS-glycoprotein, Complement C8 gamma chain, An-tithrombin-III, Serpin family A member 1, Complement factor H, C3-beta-c, Albumin, C4b-binding protein alpha chain, Alpha-1-B glycoprotein, Solute carrier family 12 member 4, and Fibronectin. Overall this report identifies new proteins involved and provides additional knowledge about the physiopathological changes related to feline obesity.

## Simple Summary

In the present study, serum samples from 20 sterilized adult domestic shorthair cats, ten with normal weight and ten with obesity, were analyzed using tandem mass tag labelling and liquid chromatography-mass spectrometry (LC-MS/MS). A total of 288 proteins were detected in the serum samples, being an abundance of 12 proteins statistically significantly different between control and obese cats. These proteins, included Ig-like domain-containing protein, Alpha-2-HS-glycoprotein, complement C8 gamma chain, antithrombin-III, and Serpin family A member 1, and are known to be involved in various psychopathological processes such as lipid metabolism, complement system, coagulation and immune response. These findings provide additional insights into the pathophysiological changes related to feline obesity, opening new avenues for research.

## 1. Introduction

Obesity is defined as the deposition of excessive adipose tissue in the body, and, currently, it is the most common disease in companion animal veterinary practice [1,2]. Obesity is caused by chronic positive energy balance due to mainly decreased exercise and increased food intake [3,4], being various environmental and genetic factors involved [5]. In cats, obesity has adverse effects on both health and welfare [6]. Its mechanical and metabolic effects predispose to several diseases, including diabetes mellitus, hepatic lipidosis, and orthopaedic, cardiac, respiratory, and urogenital disorders [7,8]. Similarly to humans, obesity in the pet population has increased over the last decades, being the prevalence of overweight and obesity in the cat population higher than 60% [9,10].

Proteomics is the analysis of the global protein profile that has emerged as a scientific technology successfully applied to several fields of human and veterinary medicine [11]. Proteomic analysis allows significant scale detection and rapid identification of proteins involved or related to disease [12]. Proteome analysis has been made in human and experimental animal models to clarify the physiopathology of obesity and its associated diseases [11,13,14]. In the same line, in canine medicine, proteomics studies have been performed in obese dogs to assess implicated metabolic pathways and to evaluate a weight-loss effect [15]. To date, in cats, proteomic studies have been performed aiming to identify serum protein markers differentiating healthy cats from cats with chronic enteropathies, chronic kidney disease, pancreatic disease, feline infectious peritonitis, and hypertrophic cardiomyopathy, among other [16,17,18,19,20]. However, to the best of the authors’ knowledge, proteomic studies have not been performed on feline obesity.

The hypothesis of this report is that proteins in serum of cats with obesity could show changes that could be detected by proteomic techniques. Based on this, the objective of this study was to evaluate the possible changes in serum proteins in feline obesity. For this purpose, serum proteomes of normal-weight cats and cats with overweight/obesity were compared. It is expected that the study of the serum proteome might provide useful information on feline obesity physiopathology and could help to identify new potential biomarkers.

## 2. Materials and Methods

### 2.1. Ethical Aspects

The study protocol was approved by the University of Murcia and Counseling of Murcia Region Ethics Committees (Number, A13170806; 2023).

### 2.2. Animals and Inclusion Criteria

Serum samples from a total of 20 adult neutered domestic shorthair client-owned cats presented at the private Veterinary clinics of the Murcia Region, Spain, for routine check-ups between 2 October 2022 and 29 July 2023, were included in the study. The inclusion criteria were the absence of abnormalities on physical examination (except overweight or obesity in the case of the obesity group) and routine hematological and biochemical analyses that included total proteins, acute phase proteins (albumin, serum amyloid A, haptoglobin, ferritin, paraoxonase 1), muscle cell damage and liver enzymes (creatinine kinase, aspartate aminotransferase, alanine aminotransferase, alkaline phosphatase, gamma-glutamyl transpeptidase, total bilirubin), lipids (cholesterol, triglycerides), amylase, urea, creatinine, and glucose, the absence of any disease or treatment in the last 6 months and negative serological titers for retroviral diseases on commercial tests (SNAP Combo FELV/FIV tests, IDEXX). Ten cats were healthy normal-weight cats (Control Group), five females and five males, with ages ranging from 1 to 8 years, and with body weight (BW) from 2.4 to 6.5 kg. The other ten cats presented overweight or obesity (Obesity Group), five females and five males, with age ranges from 1 to 12 years and BW from 4.9 to 9.0 kg.

Body condition scores (BCS) of cats were assessed using a 5-point BCS system and categorized into normal weight, controls (BCS 3) and overweight/obese (BCS 4–5) [10].

There were no statistically significant differences between the two groups in terms of sex and age, with obese cats showing higher BW and BCS (Table 1).

### 2.3. Samples

Blood samples from all cats were collected for routine hematological and biochemical analyses on the morning after an overnight (10–12 h) fast by venipuncture in the jugular vein, and placed in EDTA tubes for CBC and tubes containing a clotting accelerator (TapVal; Aquisel) for biochemistry. Surplus serum, after that required for use in routine clinical biochemical analyses, was frozen at −80 °C and used for proteomics.

### 2.4. Sample Preparation for Multiplex Proteomics

Serum protein concentrations were quantified using the BCA Protein Assay Kit from Merck (Millipore Sigma, Burlington, MA, USA), adhering to the protocol provided by the manufacturer. For digestion, 35 µg of serum protein per sample, along with an internal standard, was diluted to a final volume of 50 µL in 0.1 M triethylammonium bicarbonate (TEAB, Thermo Fisher Scientific, Waltham, MA, USA). The methods for bottom-up proteomics preparation, nano-liquid chromatography-tandem mass spectrometry (nano-LC-MS/MS), data analysis, and statistical processing as previously described [21]. Briefly, proteins were reduced with 200 mM dithiothreitol (Sigma-Aldrich, Merck KGaA, Darmstadt, Germany) at 55 °C for one hour, followed by alkylation with 375 mM iodoacetamide (Sigma-Aldrich) at ambient temperature in the dark for 30 min. The samples then underwent overnight precipitation with acetone at −20 °C. After centrifugation, the protein pellets were resuspended in 50 µL of 0.1 M TEAB. Trypsin Gold (Promega, Madison, WI, USA) was dissolved at 1 mg/mL in 0.1 M TEAB and used at a ratio of 1:35 enzyme-to-protein for digestion, which proceeded overnight at 37 °C. TMT-6-plex reagents (Thermo Scientific) were prepared as per the manufacturer’s instructions. The labeling reaction occurred at room temperature for one hour and was stopped by adding 5% (*v*/*v*) hydroxylamine (Sigma-Aldrich). Four TMT 6-plex sets were prepared, and samples were vacuum-dried for subsequent nano-LC-MS/MS analysis.

### 2.5. Nano-LC–MS/MS Analysis and Row Data Processing

High-resolution separation and detection of TMT-labeled serum peptides were carried out using the UltiMate 3000 RSLCnano system (Dionex, Germering, Germany) connected to a Q Exactive Plus Hybrid Quadrupole-Orbitrap mass spectrometer (Thermo Fisher Scientific, Bremen, Germany). Peptides, vacuum-dried beforehand, were reconstituted in a solution containing 0.1% formic acid (VWR, Avantor, Radnor, PA, USA), 2% acetonitrile (Honeywell International, Charlotte, NC, USA), and ultrapure water (Supelco, Sigma-Aldrich). The workflow included peptide trapping, desalting, nano-LC-MS/MS analysis, and Top8 data-dependent acquisition (DDA) in positive-ion mode, following the protocol established by Rešetar Maslov et al. The trap column (C18 PepMap100) and analytical column (PepMap™ RSLC C18), both sourced from Thermo Fisher Scientific, were used for peptide handling. Peptides were retained on the trap column for 12 min at a flow rate of 15 µL/min. For separation, mobile phase A consisted of 0.1% formic acid in water, while mobile phase B contained 0.1% formic acid in 80% acetonitrile. The gradient increased mobile phase B from 5% to 45% over 120 min, then rose to 90% in 2 min, was held for another 2 min, and finally re-equilibrated to 5% for 20 min at a flow rate of 300 nL/min.

The mass spectrometer was set to operate in full MS scan mode (*m*/*z* 350.0–1800.0) with a resolution of 70,000 and an injection time of 120 ms. The automatic gain control (AGC) target was 1 × 10^6^ ± 2.0 Da, with dynamic exclusion for 30 s. HCD fragmentation employed normalized collision energies (NCE) of 29% and 35%, a resolution of 17,500, and an AGC target of 2 × 10^5^. Precursor ions with unassigned charge states or those exceeding a charge state of +7 were excluded from fragmentation.

Data analysis, protein identification, and relative quantification were performed using the Proteome Discoverer software (v. 2.3, Thermo Fisher Scientific) integrated with the SEQUEST algorithm and the Felis catus database. The reference proteome FASTA file, containing 50,375 sequences, was downloaded from UniProt/SwissProt in October 2021. Proteome Discoverer parameters included allowance for two missed trypsin cleavages, a precursor mass tolerance of 10 ppm, a fragment mass tolerance of 0.02 Da, fixed carbamidomethylation (C), and dynamic modifications including oxidation (M), deamidation (N and Q), and TMT 6-plex labeling (K and peptide N-terminus). The Percolator algorithm was employed to calculate the false discovery rate (FDR), and protein identification required at least two unique peptides and an FDR of 1%. Relative quantification relied on correlating reporter ion intensities from MS/MS spectra, with normalization across 6-plexes using internal standards and within each 6-plex based on total peptide amounts.

### 2.6. Statistics for Proteomics Data

An in-house script was used for statistical analysis of quantified proteins in the open-source R software (v. 4.1.2) [22] following the procedure described previously [23] (accessed October 2021). Briefly, outliers were identified in each group for each protein using Dixon’s test (R package outliers v. 0.14 [24] and excluded if significant (*p* < 0.05). The Mann–Whitney U test was used to compare protein abundance between groups. The Benjamini–Hochberg FDR correction was applied, with significance set at FDR < 0.05, however, none of the proteins reached this criteria. Fold change was calculated as mean (cats obese)/mean (control) and expressed on a log2 scale.

### 2.7. Bioinformatic Analysis

To functionally characterise the differentially expressed proteins, gene ontology (GO) enrichment analyses were performed using the Cytoscape plugin ClueGo and its functionalities to fuse and group functionally related terms to reduce redundancy. The ontologies used were updated on 31 September 2024. Proteins and significantly enriched GO terms (*p*-value < 0.05) were represented in functionally grouped networks.

### 2.8. Validation of Albumin Results

The serum albumin, a protein that changed in the proteomic study was selected for further validation. For this purpose, serum samples from 24 cats, 12 normal-weight, and 12 overweight/obese but otherwise healthy, were analysed for albumin using a commercially available spectrophotometric assay (Albumin, Beckman Coulter Ireland Inc., Lismeehan, Ireland) in an automated analyzer (Olympus AU600, Beckman Coulter, Brea, CA, USA). The assay showed intra and interassay imprecision lower than 15% and adequate linearity after serial sample dilutions (r > 0.9).

## 3. Results

A total of 288 proteins were detected in the serum samples. Out of these, 30 proteins were detected to present statistically significant abundance between the two study groups (Table 2). However, these 30 proteins with different Uniprot accession numbers, correspond to 12 different proteins, with nine proteins showing higher abundance in cats with obesity, including Ig-like domain-containing protein, Alpha-2-HS-glycoprotein, Complement C8 gamma chain, Antithrombin-III, Serpin family A member 1, Complement factor H, C3-beta-c, Albumin, and C4b-binding protein alpha chain, and three proteins that presented lower abundance in obesity, namely Alpha-1-B glycoprotein, Solute carrier family 12 member 4, and Fibronectin. In addition, four proteins were identified as Uncharacterized protein OS = Felis catus OX = 9685 PE = 4 SV = 1, and in the four cases this protein abundance was higher in the obese cat group.

**Figure 1:**
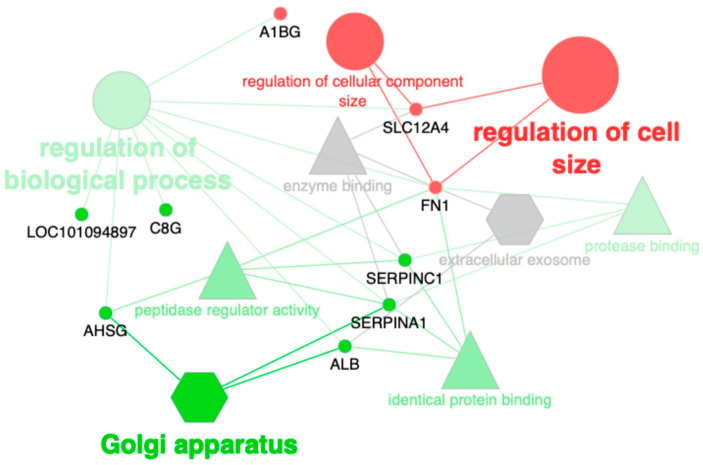
Network-like visualisation of significantly enriched GO annotations of the proteins found differentially expressed in feline serum between normal weight individuals and patients with overweight/obesity. Small, circular nodes with black labels represent proteins. Edges link proteins with their corresponding, significantly-enriched GO terms. GO terms are represented with shapes according to the ontology they belong to: ‘cellular component’ (hexagons), ‘biological process’ (circles) and ‘molecular function’ (triangles); and size that is proportional to their significance (the lower *p*-value, the larger the node size). Over-expressed proteins and their GO terms are in green, under-expressed proteins and terms are in red, and grey indicates that term is equally shared between over- and under-expressed proteins.

The GO enrichment analysis showed that serum proteins that had higher abundance in cats with obesity are significantly associated with Golgi apparatus (*p* = 0.01), regulation of biological process (*p* = 0.04) and peptidase regulator activity (*p* = 0.04). On the other hand, serum proteins that presented lower abundance in obese cats are associated with regulation of cell size (*p* < 0.01) and regulation of cellular component size (*p* = 0.01) (Figure 1; Supplementary Table S1).

### Validation of Albumin

Serum albumin concentrations were higher in cats with overweight/obesity in comparison with the normal-weight cats (*p* < 0.01) (Table 3).

## 4. Discussion

The present study aimed to evaluate differences in the serum proteome of cats with and without obesity. For this, a TMT-based quantitative approach was used, resulting in the identification of a total of 288 proteins in feline serum samples. This number is slightly higher than the reported in the same species by Yu et al. [20], who identified an average of 219 proteins using mass spectrometry-based proteomic analysis and almost double the number reported by Meachem et al. [19], who detected approximately 150 protein spots by 2D SDS-PAGE analysis. Overall, our selected methodology allowed the identification of a high number of proteins in the serum of the cats, improving the search for differently abundant proteins in the serum of cats with overweight/obesity relative to normal-weight cats.

Twelve proteins, out of a total of 288 proteins identified in feline serum in present study, showed statistically significant differences in abundance between normal-weight cats and cats with obesity. These proteins are related to lipid metabolism, complement system, coagulation and immune response. In addition, GO analysis showed that the main dysregulated GO terms were related to cell function and size regulation, as well as biological process and peptidase regulator activity alterations. In the next lines the main protein showing changes will be discussed.

Higher albumin abundance in obese cats than in normal-weight ones was found in proteomics, a finding that was confirmed by a spectrophotometric assay for albumin determination in serum. These data are in accordance with previously reported studies in obese cats [25] as well as the studies performed in humans and other animal species, such as dogs, in which obesity was related to increased albumin concentrations [26]. Regarding the mechanism of increase, it was suggested that increases in serum albumin concentrations could be due to adipose tissue hyperplasia and hypertrophy which can lead to an increase production of albumin by this tissue [27,28,29].

Herein, we describe for the first time the alteration of complement system proteins in feline obesity. In concrete, proteomic analysis revealed higher abundance of four complement system proteins—C3, C4b, C8, and CFH, in cats with obesity. These results are in accordance with a proteomic-in-gel study in dogs, where increase in complement system proteins, C2, C3, C5, C4BPA were higher in dogs with obesity-related metabolic dysfunction [30]. In human obesity, increases in complement system proteins have been described in various reports. In a previous review on humans obesity [31], increases in these proteins were positively related to body mass index, adipose tissue volume, and chronic low-level inflammation, and negatively associated with insulin sensitivity. Regarding the pathophysiology of this increase in human obesity, it has been proposed that a dysfunction of adipose tissue due to adipocyte hypertrophy may contribute to an increase in the synthesis these complement system proteins [32]. However, a direct effect of fat ingestion and circulating lipids was also reported to result in increased serum C3 levels [33]. Overall, the increased levels of complement system proteins warrants further investigations to clarify the specific pathophysiology of these alterations in the cat.

Fibronectin, which in this report was decreased in cats with obesity, is a 500–600 kDa multifunctional glycoprotein that plays a major role in cell adhesion, growth, migration, and differentiation, and it is important for processes such as cell morphology, adipogenesis, and insulin signaling of adipocytes in vitro [34]. The decrease found in our study in cats with obesity would be in line with clinical studies in humans which indicate that fibronectin levels in different adipose depots are significantly reduced in obese patients [35,36]. In addition, fibronectin concentrations were negatively correlated with leptin but positively associated with adiponectin [35].

In the present study, antithrombin III, was found to be increased in cats with obesity when compared with normal weight cats. Antithrombin III is a 464-amino-acid glycoprotein, produced by the liver, that inactivates several enzymes of the coagulation system [37]. In addition, it was shown to possess anti-inflammatory properties [38]. In dogs, decreased antithrombin activity was associated with disseminated intravascular coagulation (DIC) and mortality [39]. The presence of obesity is known to be related to the prothrombotic state in humans [40,41]. In cats, a possible hypercoagulable state in obesity was also suggested as the initiation of coagulation was faster in obese compared with lean cats and intermediate for overweight cats [42]. The increase of antithrombin III could be a reflect of the compensatory mechanism of the organism to avoid hypercoagulation, although further studies should be performed to elucidate the reason of this increase. However, caution should be taken when interpreting these results as, ideally, citrated plasma should be used for this protein determination, and, therefore, future studies are needed to confirm this observation.

Alpha-2-HS-glycoprotein, also called fetuin A, is synthesized by hepatocytes and adipocytes and is mainly involved in tissue development [43]. In our study, serum abundance of fetuin A was higher in cats with obesity. These data are in accordance with studies reported in humans, where fetuin A was shown to have a positive correlation with chronic hyperglycemia, insulin resistance, circulating lipid levels and obesity [44]. In the same manner, fetuin A was up regulated in canine DM [45]. To the best of authors knowledge there are no previous studies on fetuin A in cats, therefore, future studies are needed to evaluate the pathophysiological mechanisms related to this protein behavior in feline obesity and related pathologies.

In present study, Alpha 1-B glycoprotein and Serpin family A member 1 were more abundant in cats presenting obesity in comparison with normal-weight cats. Alpha 1-B glycoprotein is a plasma glycoprotein whose biological function is still unknown. However, in humans, its expression was elevated in different cancers, including squamous cell carcinoma of the cervix and small renal cell carcinoma, suggesting its possible role in carcinogenesis [46]. In addition, its implication in the immune system function was suggested as Alpha 1-B glycoprotein was increased in patients with chronic bronchitis [47]. On the other hand, Serpin family A member 1 is a SERPINA1 gen encoded 436 amino acid protein having serine-type endopeptidase inhibitor activity [48]. In humans, Serpin family A member 1 was associated with an increased risk of incident liver disease and was suggested to have a potential value for the diagnosis and prognosis of many human cancers being possibly involved in the immune regulation of the tumour microenvironment [49]. Therefore, it could be hypothesized that cats with obesity could be more predisposed to develop secondary pathologies such as neoplasia, disease that is known to be related to obesity [6]. In the same line, the Solute carrier family 12, member 4, a potassium/chloride transporter, was detected to be of lower abundance in cats with obesity. Although no literature is available, this alteration could be related to the increased incidence of gastrointestinal and urinary tract pathologies in cats with obesity [50].

Cats with obesity showed a higher abundance of Immunoglobulin (Ig)-domain-containing proteins. Ig-domain-containing proteins constitute the largest repertoire of surface receptors in animals having a main role in immune response [51]. The increase of these proteins in feline obesity could suggest immune system activation, and therefore, the presence of low-grade inflammatory status in these animals in accordance with previously reported studies [52].

This study has various limitations. One is that the diets that received animals included in the study were not controlled. However, the aim of this study was to gain a general picture of the animals presented at the everyday clinics. Nevertheless, it would be valuable to evaluate the possible effects of different diets on the serum proteome in cats in future studies. Secondary, the present study was a cross-sectional and cannot determine causality or long-term impacts, therefore, longitudinal studies would be needed contribute to the better understanding of the effects of obesity and obesity-management on the feline serum proteome. Also the variability of the BCS of the cats included in this report could have influenced the results. Furthermore, in addition to albumin, ideally all the other proteins showing changes should have been validated in a larger population of individuals what would have increased the reliability of the study’s results. Finally, the FDR was higher than 0.005 in all the proteins identified, this may affect the robustness of our findings and indicate the potential for false positives in our report. Therefore, further studies would be needed to explore in-depth how these proteins could behave in different obesity-related pathologies, and if these could be used for diagnosis, treatment monitoring, or prevention strategies in obese cats.

## 5. Conclusions

In conclusion, the results of this study indicate that cats with obesity present alterations in the concentrations of serum proteins with increase in Ig-like domain-containing protein, Alpha-2-HS-glycoprotein, Complement C8 gamma chain, Antithrombin-III, Serpin family A member 1, Complement factor H, C3-beta-c, Albumin, and C4b-binding protein alpha chain, and decrease in Alpha-1-B glycoprotein, Solute carrier family 12 member 4, and Fibronectin. These findings indicate the presence of alterations in lipid metabolism, complement system, coagulation and immune response associated to feline obesity.

## Figures and Tables

**Table 1:** Descriptive (median (min–max)) data of the included cats.

Variable	Control	Obesity	*p*
Sex, males/females	5/5	5/5	0.178
Age, years	2.3 (1–8)	4.5 (1–12)	0.107
BW, kg	4.3 (2.4–6.5)	6.2 (4.9–9.0)	**0.002**
BCS	3 (3–3)	4.8 (4.5–5)	**<0.001**

Data in bold highlight statistical significance.

**Table 2:** Differentially abundant serum proteins between cats with obesity (n = 10) and the control group (n = 10).

Accession (UniProt)	Protein Name	Mean Control	Mean Obese	Log2(FC)	*p* Value
P49064	Albumin OS = Felis catus OX = 9685 GN = ALB PE = 1 SV = 1	0.971	1.045	0.106	0.011
A0A2I2U7Y0	Albumin OS = Felis catus OX = 9685 GN = ALB PE = 4 SV = 3	0.97	1.045	0.107	0.009
A0A5F5XCT0	Alpha-1-B glycoprotein OS = Felis catus OX = 9685 GN = A1BG PE = 4 SV = 1	1.091	0.95	−0.200	0.007
M3W0W4	Alpha-1-B glycoprotein OS = Felis catus OX = 9685 GN = A1BG PE = 4 SV = 3	1.09	0.951	−0.197	0.007
A0A5F5XVZ8	Alpha-2-HS-glycoprotein OS = Felis catus OX = 9685 GN = AHSG PE = 4 SV = 1	0.905	1.135	0.327	0.017
A0A5F5Y4L1	Alpha-2-HS-glycoprotein OS = Felis catus OX = 9685 GN = AHSG PE = 4 SV = 1	0.879	1.151	0.389	0.009
A0A337SD37	Alpha-2-HS-glycoprotein OS = Felis catus OX = 9685 GN = AHSG PE = 4 SV = 2	0.879	1.151	0.389	0.009
M3WLL8	Antithrombin-III OS = Felis catus OX = 9685 GN = SERPINC1 PE = 3 SV = 1	0.894	1.016	0.185	0.023
A0A5F5XER5	C3-beta-c OS = Felis catus OX = 9685 PE = 4 SV = 1	0.908	0.984	0.116	0.045
A0A5F5XIJ2	C3-beta-c OS = Felis catus OX = 9685 PE = 4 SV = 1	0.908	0.984	0.116	0.045
A0A5F5XZV0	C3-beta-c OS = Felis catus OX = 9685 PE = 4 SV = 1	0.908	0.984	0.116	0.045
A0A5F5Y2A2	C3-beta-c OS = Felis catus OX = 9685 PE = 4 SV = 1	0.908	0.984	0.116	0.045
A0A5F5Y2U5	C3-beta-c OS = Felis catus OX = 9685 PE = 4 SV = 1	0.908	0.984	0.116	0.045
A0A5F5Y592	C3-beta-c OS = Felis catus OX = 9685 PE = 4 SV = 1	0.908	0.984	0.116	0.045
M3WJK3	C4b-binding protein alpha chain OS = Felis catus OX = 9685 GN = C4BPA PE = 4 SV = 4	0.939	1.008	0.102	0.027
M3VUN7	Complement C8 gamma chain OS = Felis catus OX = 9685 GN = C8G PE = 3 SV = 1	0.913	1.067	0.225	0.002
A0A5F5XM81	Complement factor H OS = Felis catus OX = 9685 GN = LOC101089505 PE = 4 SV = 1	0.914	1.032	0.175	0.001
A0A5F5XMT8	Complement factor H OS = Felis catus OX = 9685 GN = LOC101089505 PE = 4 SV = 1	0.94	1.031	0.133	0.023
A0A5F5XS16	Complement factor H OS = Felis catus OX = 9685 GN = LOC101089505 PE = 4 SV = 1	0.944	1.031	0.127	0.035
A0A5F5XWF0	Complement factor H OS = Felis catus OX = 9685 GN = LOC101089505 PE = 4 SV = 1	0.944	1.031	0.127	0.035
A0A337S3R0	Complement factor H OS = Felis catus OX = 9685 GN = LOC101089505 PE = 4 SV = 2	0.951	1.045	0.136	0.023
A0A337SWF2	Fibronectin OS = Felis catus OX = 9685 GN = FN1 PE = 4 SV = 1	1.39	0.827	−0.749	0.043
A0A2I2USM8	Ig-like domain-containing protein OS = Felis catus OX = 9685 PE = 4 SV = 2	0.785	1.044	0.411	0.028
A0A5F5XW04	Serpin family A member 1 OS = Felis catus OX = 9685 GN = SERPINA1 PE = 3 SV = 1	0.941	1.08	0.199	0.003
M3WCX1	Serpin family A member 1 OS = Felis catus OX = 9685 GN = SERPINA1 PE = 3 SV = 2	0.964	1.074	0.156	0.011
M3WNL1	Solute carrier family 12 member 4 OS = Felis catus OX = 9685 GN = SLC12A4 PE = 3 SV = 3	1.119	0.941	−0.250	0.007
A0A337SAI9	Uncharacterized protein OS = Felis catus OX = 9685 PE = 4 SV = 1	1.372	0.768	−0.837	0.005
A0A337S7U4	Uncharacterized protein OS = Felis catus OX = 9685 PE = 4 SV = 2	1.372	0.768	−0.837	0.005
A0A337S7W6	Uncharacterized protein OS = Felis catus OX = 9685 PE = 4 SV = 2	1.372	0.768	−0.837	0.005
A0A337SV91	Uncharacterized protein OS = Felis catus OX = 9685 PE = 4 SV = 2	1.372	0.768	−0.837	0.005

FC: fold change.

**Table 3:** Sex, body condition score (BCS), age, and albumin (laboratory reference range) data of the animals included in the validation study of the albumin results.

Group	Animal	Sex	BCS	Age	Albumin (2.5–3.6)
Normal-weight	1	MC	5	6	3
	2	HC	5	1	3.1
	3	HC	5	7	3.1
	4	H	5	1	3.0
	5	HC	5	2	3.1
	6	MC	5	6	3.0
	7	HC	5	3	3.1
	8	MC	5	2	3.2
	9	HC	5	6	3.6
	10	MC	5	12	3.3
	11	MC	4	22	3.6
	12	HC	5	1.5	3.6
Median (25–75%)				4.5 (1.6–6.8)	3.1 (3.0–3.5)
					
Overweight-obese	1	HC	6	4	3.4
	2	MC	6	2	3.1
	3	HC	6	1	3.3
	4	H	6	1	3.6
	5	HC	7	4	3.4
	6	MC	8	8	3.4
	7	MC	7	8	4
	8	HC	7	7	3.5
	9	MC	5	8	3.7
	10	MC	5	7	4.1
	11	MC	8	8	3.6
	12	HC	5.5	8	4.3
Median (25–75%)				7.0 (2.5–8.0)	3.6 (3.4–3.9)
	*p*			0.396	0.005

## Data Availability

The data are available upon reasonable request.

## Supplementary Materials

The following supporting information can be downloaded at: https://www.mdpi.com/article/10.3390/ani15010091/s1, Table S1: ClueGO Results.
